# Adding Dimensions to the Analysis of the Quality of Health Information of Websites Returned by Google: Cluster Analysis Identifies Patterns of Websites According to their Classification and the Type of Intervention Described

**DOI:** 10.3389/fpubh.2015.00204

**Published:** 2015-08-25

**Authors:** Mubashar Yaqub, Pietro Ghezzi

**Affiliations:** ^1^Brighton and Sussex Medical School, Brighton, UK

**Keywords:** Internet, IQ, websites, migraine, information, online

## Abstract

**Background and aims:**

Most of the instruments used to assess the quality of health information on the Web (e.g., the JAMA criteria) only analyze one dimension of information quality (IQ), trustworthiness. In this study, we analyzed the type of intervention that websites describe, whether supported by evidence-based medicine (EBM) or not, to provide a further dimension of IQ, accuracy, and correlated this with the established criteria.

**Methods:**

We searched Google for “migraine cure” and analyzed the first 200 websites for: (1) JAMA criteria (authorship, attribution, disclosure, currency); (2) class of websites (commercial, health portals, professional, patient groups, no-profit); and (3) type of intervention described (approved drugs, alternative medicine, food, procedures, lifestyle, drugs still at the research stage). We used hierarchical cluster analysis to identify different patterns of websites according to their classification and the information provided. Subgroup analysis on the first 10 websites returned was performed.

**Results:**

Google returned health portals (44%), followed by commercial websites (31%) and journalism websites (11%). The type of intervention mentioned most often was alternative medicine (55%), followed by procedures (49%), lifestyle (42%), food (41%), and approved drugs (35%). Cluster analysis indicated that health portals are more likely to describe more than one type of treatment while commercial websites most often describe only one. The average JAMA score of commercial websites was significantly lower than for health portals or journalism websites, and this was mainly due to lack of information on the authors of the text and indication of the date the information was written. Looking at the first 10 websites from Google, commercial websites are underrepresented and approved drugs overrepresented.

**Conclusion:**

Analyzing the type of therapies/prevention methods provides additional information to the trustworthiness measures, such as the JAMA score, and could be a convenient and objective indicator of websites whose information is based on EBM.

## Introduction

As more patients search for health information on the Internet, many studies have analyzed the quality of health information available on the web for different pathological conditions. There is a concern that, because the Internet is practically not controlled or regulated, this might expose the public to misinformation, particularly those with low information literacy, which is the ability to critically appraise the information (1, 2). This could potentially result in patients turning to non-approved therapies, whose efficacy (and risks associated) has not been scientifically proven. Furthermore, in recent years, there have been an increasing number of commercial websites selling counterfeit medicines, with additional risks for vulnerable patients (3).

Although there are a number of specialized health websites, most patients will use generic search engines, such as Google, to search for health-related information (4), and several studies have tried to address the quality of health information available on the Internet using various methods.

The most used are the Health-on-the-Net [HON (5)] seal of approval, the JAMA criteria (6), and the DISCERN criteria (7). The latter two are, in fact, instruments to quantitatively measure the information quality (IQ) by assigning a score based on the website matching some requirements. In particular, these criteria consider whether a website provides information about authorship, ownership/financial interests, advertising, contact details, or date of update (currency). Another parameter that is also evaluated when assessing health information is its readability. For health information to be accessible to lay people, it has to be comprehensible by the average reader, and it was suggested that a patient information leaflet should be aimed at a reading ease of sixth grade in order to be understandable by 75% of the population (8). Studies have shown that the average reading level of health websites is between 10th and 13th grade (9, 10), and thus may not be accessible to individuals with lower literacy. Of note, readability and the trustworthiness indicators above do not always correlate, and one study reported that readability might be lower in websites displaying health seals of approval (11).

All of the criteria for evaluating health websites mentioned above do not take into consideration the content of the website. Therefore, they are mainly evaluating the transparency/trustworthiness of the website rather than the overall IQ, in that they do not consider the content of the site and whether the information provided is scientifically correct and evidence-based.

There are several dimensions of IQ as originally described by Wang and Strong in their seminal study (12). The study identified almost 200 data quality attributes and 18 dimensions of IQ. This and other studies group the various attributes of IQ in four categories of IQ: accessibility IQ, representational IQ (that includes understandability, format, appearance), contextual IQ (including completeness and timeliness), and intrinsic IQ (that include accuracy, correctness, and objectivity) (13). In the context of health information, probably intrinsic IQ components should include the correctness of the description of the scientific basis of the information described, that would be missing, for instance, in a website reporting that AIDS is not due to a viral infection. Assessment of these intrinsic dimensions of IQ will require an in-depth analysis of the content to be done by a panel of experts. A few studies have analyzed the content of the websites. For instance, Peterlin et al. analyzed the IQ available on cluster headache in 40 websites found using the search engine, MetaCrawler (14). In that study, the authors used a scoring system for the technical component that evaluated the correctness of the information on aspects, such as epidemiology, risk factors, diagnosis, pathophysiology, treatment, and prevention. A similar approach has been used to assess the IQ in 114 websites returned by MetaCrawler or MSN searching for retinopathy of prematurity (15).

In this study, we aim analyzing the type of treatment reported, an alternative, and simpler strategy to add an intrinsic quality dimension to the health IQ, beyond the first layer of contextual and representational IQ measured by the JAMA score.

We searched the words “migraine cure” because we aimed at looking at the information that would be returned from a search done by a layperson. In fact, studies have shown that the quality of information varies with the search term used, that is the more sophisticated the search term, the higher the IQ (16, 17). It should be noted, however, that we were more interested in setting up a methodology to study the correlations between classes of websites, type of recommendation, and the JAMA criteria as a trustworthiness index, and we did not assign a particular importance to the search terms used.

We selected the search engine, Google, because it is the most popular search engine, and, as a reference, one specialized health portal (the US Government Medline Plus). We analyzed the first 200 hits and classified the type of websites according to their affiliation (i.e., if they were commercial, professional, health portal, journalism, patient group, non-profit, or other). We also analyzed the text of the websites and classified the treatments/interventions described (whether approved drugs, alternative medicine, lifestyle or nutritional advice, etc.). We also used an established measure of the health IQ of websites (the JAMA criteria).

## Materials and Methods

We searched for websites containing information relating to management of migraine. The search was conducted between December 2013 and January 2014, using google.com (US) clearing cookies and using the “private browsing” of Mozilla Firefox browser to prevent, as much as possible, personalized results due to previous browsing history. We are aware that the search results may still be influenced by the detection of the geographical location via the IP address.

Google search found 6,040,000 hits and we considered the first 200. Of those, 198 were accessible (thus excluding non-functioning links; denied direct access through password requirement, payment or subscription; not written in English) and were used in the subsequent analysis. We did not take into account the websites marked “advertisement” that Google shows before the actual search results.

**Figure 1:**
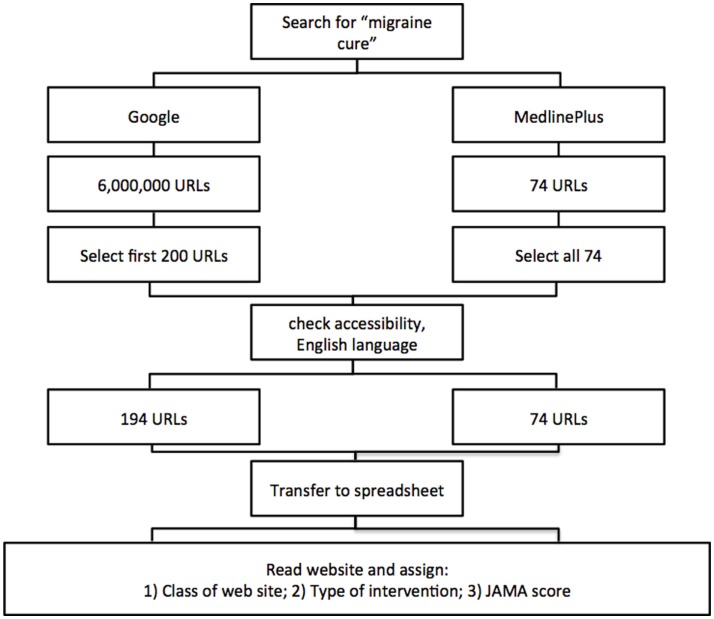
**Flow chart of data collection and analysis**.

MedlinePlus returned 74 hits that were all accessible and included in the analysis. The flow chart describing how the data were collected and processed is shown in Figure 1. Website links were transferred into a spreadsheet from Google using the SEOquake tool. For MedlinePlus, links were manually transferred into a spreadsheet.

Websites were classified in accordance to their affiliation as being professional, patient group, commercial, health portal, non-profit, journalism, or others, as summarized in Table 1. Two researchers independently classified the websites and their findings were compared. For those cases where there was no agreement, the website was revisited by both researchers and a consensus achieved by discussion.

**Table 1:** **Classes of websites**.

Affiliation	Description	Examples
Professional (P)	Website created by a person or organization with professional knowledge of the information, e.g., government, institutions, libraries, universities, publishers, online scientific journals, and other educational institutions	nhs.ukn
		umn.edu
		ninds.nih.gov
Commercial (C)	Websites that buy, sell, or provide a service for a fee, e.g., profit organizations	lipigesic.com/
		migracap.co.uk/
		walgreens.com/
Health portal/blog (HP)	Website or search engine with health information on a verity of health topics, e.g., health blogs	joybauer.com/
		mayoclinic.com/
		healthline.com/
Patient group (PG)	Websites targeted at patients or created by patients, e.g., patient blogs, patient forums, chat rooms, and support groups	curetogether.com
		curezone.org/forums
		myhomeremedies.com
Journalism	Websites primarily broadcasting news online, providing information relating to health topics	foxnews.com
		bbc.co.uk
		philly.com/
Non-profit (NP)	Websites providing information for educational or charitable reasons with no financial beneficiaries, e.g., charitable organizations	wikipedia.org
		migrainetrust.org
		migraine.ie
Other (O)	Websites, which do not fit into any of the other affiliations. Includes social networking sites	facebook.com
		twitter.com

There were 10 disagreements on 196 websites (95% agreement). Calculating inter-rater reliability for two coders using Recal2 (18) showed that the highest disagreement was on “Other” websites (Cohen’s kappa coefficient, −0.01; six disagreements) followed by “Professional” websites (Cohen’s kappa coefficient, 0.76; three disagreements), and “Patients Groups” (Cohen’s kappa coefficient, 0.86; three disagreements). All other classes of websites (Commercial, Health Portal, Journalism, No-profit) showed higher agreement rates, with Cohen’s kappa coefficient >0.93. In the case of Health Portals, there were five disagreements but, because of the large number of websites in this class, Cohen’s kappa coefficient was 0.95. There was no disagreement on the type of intervention described as that was less subjective as was simply based on an intervention being mentioned in the text.

Websites were then classified according to the different types of intervention they indicated as described in Table 2. One website could mention more than one intervention. If a website gave a link to a different page of the same website, this information was also analyzed. However, if the link was given to an external site, this information was dismissed. If a website mentioned an intervention only to state that it is not effective, then the information was dismissed. Therefore, a website was tagged for a type of intervention if either it mentioned the intervention being an effective one, or listed it as one of the possible interventions without further comments.

**Table 2:** **Intervention groups**.

Intervention	Description
Approved drug	Pharmacological therapy validated for a therapeutic use by the FDA or the British National Formulary
Alternative medicine	Therapies that are not based on scientific evidence. These include homeopathy, herbalism, naturopathy, and crystal healing
Food	Recommendation of food for management of migraine. These include coffee, lavender tea, ginger, and honey
Procedure and devices	Recommendation of any procedure or use of a device for management of migraine. These include surgery, biofeedback, and migraine cap
Lifestyle and triggers	Altering lifestyle factors and/or avoidance of triggers of migraine (e.g., recommending regular sleeping or avoiding alcohol)
Research drug	Pharmacological therapy, which is still in research stages and not yet approved for the use of migraine (e.g., lidocaine, calcitonin gene-related peptide receptor antagonists)
No information given	Gave no information on how to manage migraine

We then gave each website a score according to the JAMA criteria (6, 19, 20). For this purpose, websites were analyzed for the following information: (1) authorship (identification of authors/contributors); (2) attribution (references listing sources of information); (3) disclosure (of ownership, advertising, conflict of interests); and (4) indication of date content was posted or updated. For each of these four criteria, we assigned a score of 1 if the information was present, or 0 if absent or unclear. If the information was not available on the initial website information, then the three-click rule was used. The three-click rule is an unofficial website navigation rule that suggests information should be accessible within three clicks (21). In previous studies, a website scoring a mean JAMA score of 3 or above has been suggested to be of high quality (19, 22).

The Kruskal–Wallis test was used for multiple comparisons of non-parametric variables, followed by Dunn’s test, using GraphPad Prism software (GraphPad Prism Software Inc., La Jolla, CA, USA). The Mann–Whitney test was used where there were two independent groups. When indicated, contingency tables were analyzed using a one-tailed Chi-square test for non-parametric data. Hierarchical cluster analysis was performed using the Genesis software (http://genome.tugraz.at/genesisclient/genesisclient_description.shtml) (Version 1.7.6 for Mac OSX).

Because websites URLs are not permanent to ensure that the reader will be able to see examples of the search results, the top 10 URLs returned in each of the 5 Google SERPs listed in Tables S1 and S2 in Supplementary Material were archived. For this purpose, we used WebCite^®^, an on-demand archiving system for webreferences and the archived URL is provided next to the original URL. Two webpages, all from the domain, www.health.com, were indicated as not available (n/a) as they could not be archived, presumably because either the site in question refuses connections by crawling robots or is inaccessible from the WebCite network.

## Results

### Distribution of websites

The 198 websites returned by Google were analyzed by their affiliation and the type of intervention they describe. The raw data, with the list of websites URLs and how they were classified in terms of class of websites, type of intervention, and JAMA scores are provided in Table S1 in Supplementary Material. As shown in Table 3, commercial websites and health portals made up for 75% of the total, in contrast with the 74 returned by MedlinePlus, where professional websites accounted for nearly 90% of the hits returned.

**Table 3:** **Distribution of websites generated from search in Google or MedlinePlus according to their affiliation**.

Affiliation	Google (%)	MedlinePlus (%)
C (commercial)	31	
HP (health portal)	44	1
J (journalism)	11	
NP (non-profit)	4	
P (professional)	3	89
PG (patient group)	6	10
O (other)	1	

*Data represent the percentage of websites in each affiliation. Total number of websites was 198 for Google and 74 for MedlinePlus*.

Table 4 shows the distribution of websites according to the type of intervention described. Of the 198 websites returned by Google, 184 could be assigned to an “intervention” group. However, of the 74 websites returned by MedlinePlus, 70% did not mention an intervention and could not be considered for this type of analysis. The largest proportion of websites returned by Google dealt with alternative medicine (55%) followed by procedures and devices (49%). The total for Google adds up to more than 100% because, many websites mentioned more than one type of intervention.

**Table 4:** **Distribution of websites generated from search in Google or MedlinePlus according to the intervention indicated**.

Intervention	Google (%)	MedlinePlus (%)
Approved drug	35	23
Alternative medicine	55	8
Food	41	3
Procedures and devices	49	7
Lifestyle and triggers	42	11
Research drugs	3	3
No information given	5	70

*Data represent the percentage of websites mentioning a type of intervention. Total number of websites was 198 for Google and 74 for MedlinePlus*.

### Cluster analysis of website patterns

**Figure 2:**
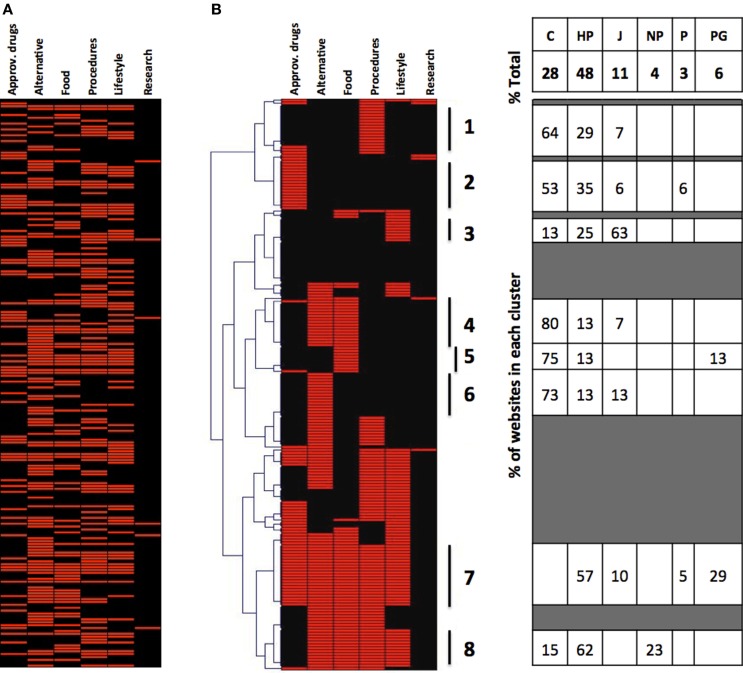
**Cluster analysis of the websites returned by Google**. **(A)** Type of intervention mentioned by the 198 websites (in alphabetical order by website URL). **(B)** Hierarchical cluster analysis on websites from **(A)**, clustered by type of intervention. The table on the right shows the composition by class of websites of the eight clusters identified in this figure.

We then decided to analyze whether groups of websites could be identified based on the type of interventions they describe and taking into account the fact that most of them will describe more than one type of intervention. The type of data visualization used above does not allow a detailed analysis of co-occurrence of intervention mentioned. Therefore, we have used a graphic representation where each website is assigned a value of 1 for each type of intervention mentioned. Each website is listed in one row and the type of intervention in a column. The value 1 is then represented in red and the visual representation is shown in Figure 2A, where one can appreciate that some websites mention more than one intervention and some only one.

We wanted to see, using this type of visual representation, whether websites mentioned or excluded specific interventions and whether this correlated with the class of website. We thus first performed a hierarchical cluster analysis of the websites according to the intervention they mention, and the results are presented in Figure 2B. In the left part of the figure, it can be seen that we can identify clusters of websites that describe only procedure and devices (cluster 1), only approved drugs (cluster 2), only lifestyle (cluster 3), only alternative medicine (cluster 6), and websites that describe all possible interventions (cluster 7) except for the experimental drugs that were mentioned in very few websites. While there were several websites (cluster 8) that described multiple interventions excluding approved drugs, only one website described multiple interventions excluding alternative medicine.

This type of visualization allowed us to then analyze the composition, in terms of the different classes of websites, of these clusters, and the results are shown in the left side of Figure 2B.

The commercial websites preferentially mention only one intervention. In fact, while commercial websites are 28% of the total, they are 75% of those mentioning only nutrition, 73% of those mentioning only alternative medicine, 64% of those mentioning only procedures and devices. However, there are no commercial websites among those mentioning all possible indications (cluster 7), and only 15% in those mentioning all interventions excluding approved drugs (cluster 8). Health portals, which are 48% of the total, were overrepresented among the websites reporting several treatment options (clusters 7 and 8). Journalism websites, which represent 11% of the total, are prevalent among those that only mention “lifestyle” interventions (cluster 3, 63%) and never mention all the possible interventions (cluster 7). It is difficult to comment on the other classes of websites due to their small number. Likewise, a cluster analysis of the MedlinePlus did not provide much information due to the small number of websites, of those returned, that describe an intervention (not shown).

The fact that commercial websites preferentially describe only one type of intervention is also evident by calculating the mean number of different types of intervention described by the different classes of websites (Table 5).

**Table 5:** **Average number of intervention types described by the different classes of websites**.

Website class	No. of treatments indicated	*n*
Commercial	1 ± 1^ab^	60
Health portals	3 ± 1.4^a^	87
Journalism	2 ± 1.4	22
No-profit	3 ± 1.3	8
Professional	3 ± 2.1	6
Patient groups	4 ± 1.9^b^	11

*Data are mean ± SD. *n* is the number of websites in each class. Values bearing the same symbol are significantly different from each other*.

*^a^*P* < 0.0001; ^b^*P* < 0.005*.

*Kruskal–Wallis test was used for multiple comparisons of non-parametric variables, followed by Dunn’s test*.

It can be seen that the average number of treatments described by the website is 1 in the commercial websites, followed by journalistic sources with an average of 2, health portals, non-profit, and professional websites with an average of 3, while patient group websites are those describing a wider picture of treatments (an average of 4). Commercial websites describe significantly less treatment options than health portals or patient groups.

### Distribution of classes of websites and types of intervention in the ranking of google search: Analysis of the first 10 websites returned

We wanted to investigate whether the top 10 websites returned by Google were following a different pattern in terms of class of website or type of intervention described. As shown in Table 6, there were significantly less commercial websites (0/10) in the top 10 then in all 198 websites (60/198). No other significant difference was observed for any of the other classes of websites. Thus, commercial websites are underrepresented in the top 10 results. Table 6 also shows the occurrence of the different types of intervention in the top 10 websites and in the total number of websites. The only significant difference found was that approved drugs are overrepresented in the 10 top ranking websites.

**Table 6:** **Distribution of classes of websites and types of intervention in the ranking of Google search**.

Website class	Number in top 10	Number in total	Type of intervention	Number in top 10	Number in total
Commercial	0^a^	60^a^	Approved drug	8^b^	70^b^
Health portals	5	89	Alternative medicine	5	109
Journalism	2	22	Food	8	81
No-profit	1	8	Procedures and devices	5	98
Professional	1	6	Lifestyle and triggers	8	84
Patient groups	1	12	Research drugs	2	6

*Occurrence of classes of websites and type of intervention they describe in the first 10 hits and in the total of 198 websites*.

*Values bearing the same letter are significantly different from each other by Chi-square test*.

*^a^*P* < 0.05; ^b^*P* < 0.05*.

### Analysis of trustworthiness (JAMA scores) of the different classes of websites

In this part of the study, we wanted to assess whether different classes of websites or websites describing different types of interventions differed for their JAMA score, as a recognized measure of website quality/trustworthiness.

Overall, there was a small but significant difference (*P* < 0.02 by Student’s *t*-test) in the average JAMA score of websites returned from Google (1.9 ± 0.8; 27% having a score ≥3) or MedlinePlus (2.1 ± 0.6; 19% having a score ≥3). The number of websites with a JAMA score ≥3 was slightly higher with Google (Google, 55/198, 27%; MedlinePlus, 14/74, 19%) but the difference was not statistically significant (Chi-square without Yates correction gave a one-tailed *P* value of 0.0675). The distribution of JAMA scores was different, and while in MedlinePlus 72% of the websites scored 2, and 19% scored 3, Google had a broader distribution, with 39, 33, and 27%, scoring 1, 2, and 3, respectively).

We then analyzed the JAMA score of websites returned from Google according to their class and the type of intervention, and the results are reported in Table 7. It can be seen that there are significant differences in the mean JAMA score of different classes of websites, with journalistic websites scoring the highest and commercial websites the lowest. This is also evident if we look at the percentage of websites that have a JAMA score of ≥3, where journalism websites score the highest, followed by health portals, and commercial websites the lowest. It is difficult to comment on classes of websites with 10 or less websites.

**Table 7:** **Mean JAMA score of websites by class and type of intervention**.

Website class	Mean JAMA score	% >3	Type of intervention	Mean JAMA score	% >3
Commercial	1.4 ± 0.7^ab^ (60)	10	Approved drug	2.09 ± 0.8 (70)	31
Health portals	2.1 ± 0.9^ae^ (87)	38	Alternative medicine	1.84 ± 0.84 (109)	24
Journalism	2.6 ± 0.5^bcd^ (22)	64	Food	1.96 ± 0.83 (21)	26
No-profit	1.1 ± 0.6^ce^ (8)	0	Procedures and devices	1.82 ± 0.82 (22)	22
Professional	2.3 ± 0.5 (6)	33	Lifestyle and triggers	1.99 ± 0.87 (26)	31
Patient groups	1.5 ± 0.5^d^ (11)	0	Research drugs	2.5 ± 0.55 (6)	50

*Data are the mean JAMA score ± SD; the number of websites in each group is indicated in parentheses. Values bearing the same letter are significantly different from each other*.

*^a^*P* < 0.0001; ^b^*P* < 0.0001; ^c^*P* < 0.0005; ^d^*P* < 0.01; ^e^*P* < 0.05*.

*Kruskal–Wallis test was used for multiple comparisons of non-parametric variables, followed by Dunn’s test*.

When we look at the JAMA score of websites classified by types of intervention, approved drugs score slightly higher but the differences are small and not statistically significant. Again, it is difficult to comment on the high score of websites that mention research drugs, as there are only 6.

Because MedlinePlus returned only two types of websites, and few of them indicated an intervention, this analysis has not been performed on those websites.

To identify the reasons for the different JAMA scores in the different classes of website described in Table 7, we have disaggregated the JAMA score in its four components and have analyzed them in the different classes of websites (Table 8). From this is clear that most websites met the “disclosure” criteria, and very few the “attribution.” The two criteria for which commercial websites and no-profit ones differed the most from the ones having a higher JAMA score (health portals and journalism) were “authorship” and “currency.”

**Table 8:** **JAMA score components in the different classes of websites**.

Website class	Authorship	Attribution	Disclosure	Currency
Commercial	17	5	100	22
Health portals	48	7	100	56
Journalism	64	5	100	95
No-profit	0	13	88	13
Professional	33	17	100	83
Patient groups	9	0	100	45

*Data indicate the percentage of websites in each class that met the specific criteria*.

**Figure 3:**
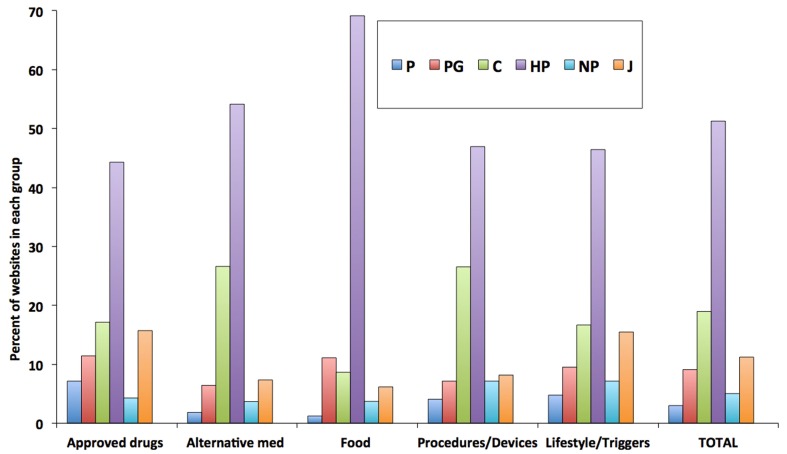
**Composition of websites mentioning different types of intervention**. Data are expressed as a percentage of the total number of websites mentioning a type of intervention. Data labels are shown above each bar. Research drugs as type of intervention are not included because of the small number of websites (*n* = 4).

We then analyzed the distribution of intervention groups by class of websites. As shown in Figure 3, it is clear that the pattern of classes of websites is similar across all the types on intervention mentioned, i.e., there is not a type of intervention that is preferentially described by a specific class of websites. Possibly, there is a preference toward alternative medicine and procedure/devices in commercial websites, and for nutritional interventions in health portals. Of note, the pattern cannot be compared with that given in Table 3 because website can mention multiple types of interventions and an average value, obtained by adding up the values for all the interventions, is shown for comparison.

## Discussion

The present study extends our previous approach of assessing the quality of the health information of websites returned by search engines using a medical search term. In the previous work, we analyzed different classes of websites using instruments (HON code, JAMA score) that measure the trustworthiness of a website rather than the quality of the information provided. We have analyzed the websites returned by a Google search in terms of the type of intervention indicated in their content. Of course, we cannot make conclusion from our findings that could be extended to any search query. Also, results returned by Google will change with time, location, and search history. Nevertheless, the 200 websites returned represent a good sample of migraine-related websites so that some conclusion can be drawn on the usefulness of the methodology described here.

The approach used here is unbiased, and does not provide an absolute indicator of “health IQ.” However, seen from the perspective of evidence-based medicine (EBM), one should consider a website of higher quality if it points to a drug approved by a regulatory agency, and that has gone through EBM criteria for its approval, rather than to, for instance, crystal healing.

In this respect, it is important to note that, as shown in Table 7, a trustworthiness score, such as the JAMA score, is not predictive of whether a website will promote the use of alternative medicine rather than that of an approved drug, confirming that the type of intervention and the criteria used in the JAMA score are assessing different dimensions of IQ. This confirms conclusions made from a larger study on over 300 web pages providing information on breast cancer, where the JAMA score was found not to predict whether the information provided was scientifically accurate (23).

Commercial websites, no-profit organizations and patient groups have a lower JAMA score lower than health portals, professional, or journalism websites. This is exactly the same pattern that we previously reported in a study on health information on diabetic neuropathy, independently on the search engine used, whether it was Google, Yahoo, Bing, or Ask (24).

One recommendation that can be made from this observation is that owners/publishers of commercial, patient group, and no-profit websites try to improve their trustworthiness, which could often be achieved by providing simple information, such as the author of the text, and date of last update.

Another pattern that is evidenced in the present study (particularly in the cluster analysis and in Table 5) is that commercial websites will often describe only one type of intervention. This comes as no surprise and it was probably expected that a website, www.tylenol.com, describes only acetaminophen; it is probably a very good source of information on how to use this drug. However, for a patient searching the Internet on how to cure their disease, it is important to have websites that mention different options, possibly with a critical analysis, or at least a description, of their respective benefits, risks, and the scientific evidence for their efficacy. This might identify criteria to be considered in evaluating health websites, inclusivity as opposed to exclusiveness. Clearly, including various intervention options, it may be desirable or not depending on the purpose of a website, but it may be helpful to direct patients toward health portals if they want to have a general idea of the different options and to commercial websites if they seek information on one specific type of intervention.

An important question in studies of this kind is whether it is relevant to analyze 200 websites returned by Google. In fact, many studies have shown that the user directs most of the attention to the first items in the search list (25) and a study in patients searching health-related information found that they will usually look at the first 10 websites returned by the search engine (2). The difference in the pattern observed for all 200 websites and the top 10 is most interesting. Contrary to common belief that for-profit search engines, like Google, will preferentially return commercial websites, we found, as shown in Table 6, that these were in fact underrepresented, to a statistically significant extent, in the top 10 results. Of course, this may depend on the search terms, or the disease condition we search for. We have reanalyzed the raw data from our previous study where we searched the term “diabetic pain” using Google and analyzed 200 websites returned (24), and found that also in that dataset, commercial websites are underrepresented in the first 10 hits. In fact, in that study, the total number of commercial websites was 42 but they were not present in the first 10 hits. Also in that case, commercial websites were significantly underrepresented. To assess how much this depends on the terms used in the query, we performed, during the revision of this manuscript, 4 different searches on “migraine cure,” “migraine medicine,” “migraine treatment,” and “migraine therapy” and looked at the top 10 websites returned by Google. As shown in Table S2 in Supplementary Material, there were no websites classifiable as “Commercial” in any of these SERPs. Clearly, we cannot generalize and the distribution of websites returned will vary with the terms used in the queries. It would be interesting to characterize the pattern of websites returned by different search queries, possibly analyzing also existing, published data. We reanalyzed the data reported in an IQ study on kidney transplant for which a list of 94 websites was reported. In that study, commercial websites were 25% of the total, but none was in the top 10 returned by Google (26).

Also, it is often suspected that the Internet would generally point the patient toward alternative medicine approaches rather than medicinal products whose approval is based on EBM criteria. However, the data reported here show that, although among the total 200 websites analyzed that alternative medicine interventions are mentioned more frequently than approved drugs (55 vs. 35%), this is not true in the first 10 items returned by Google, where approved drugs are significantly overrepresented.

Of course, there are other features of the websites that should be taken into account. The fact that a website is returned in a Google search does not mean that it will be read. That depends on several issues including attractiveness, readability, and various aspects of the website’s design and content, that would require, in addition to the readability test mentioned above, using eye-tracking analysis (27).

More importantly, we need to stress once again that these results were obtained with a specific query. Anyone who had a chance of searching the Internet for health information knows how often low-quality websites pointing the layperson toward treatments, or strategies for prevention, that are not evidence-based, are found in the top results. Ideally, performing a large number of searches on different health-related queries would point at where misinformation, or disinformation, makes it to the top of the search. Clearly, this is a larger project that would require machine processing rather than individual scoring. The use of the type of intervention described here is something that could be implemented by defining list of keywords that using automated machine learning. This study shows that analyzing websites for the type of intervention they describe could provide other dimensions of health IQ in addition to those described by the JAMA score. In fact, at least within the sample of 200 websites retrieved here, the JAMA score did not differentiate between websites describing approved drugs from those describing alternative medicine approaches.

Performing a cluster analysis of websites, based on their classification and the intervention described, can identify patterns of websites pointing patients toward one or more treatments. In the specific example of health-related query studied here, we were surprised that commercial websites ranked low in the search list. In terms of the type of intervention recommended, complementary/alternative medicine occurred more often than approved, EBM-based drugs, but the latter were more frequent in the top 10 results, possibly due to the intrinsic higher IQ features of these websites.

It would be important to apply this type of analysis over a wide range of search terms and disease conditions and health topics in order to identify areas that are more at risk of directing patients toward non-EBM types of intervention, and thus potentially pose problems to public healthcare systems and health insurance companies. This would indicate where policy makers and professional organizations should concentrate their effort to inform patients on the benefits, risk, and scientific basis of existing therapies.

### Limitations

The results of the study are specific for one specific query on one health topic. In particular, the observation that commercial websites are under-represented in the first 10 results, and that health portals are the most frequent class of websites and alternative medicine the most frequently described type of intervention may be specific to this topic; searches in other health domains may give completely different results. Additional studies on different health topics will be needed to assess the usefulness of the proposed methodology (classification by type of intervention and cluster analysis). Finally, the type of classification we have used (both in terms of website classes and types of interventions) may need to be tailored to the specific research questions.

## Author Contributions

MY and PG both analyzed the data and wrote the paper.

## Conflict of Interest Statement

The authors declare that the research was conducted in the absence of any commercial or financial relationships that could be construed as a potential conflict of interest.

## Supplementary Material

The Supplementary Material for this article can be found online at http://journal.frontiersin.org/article/10.3389/fpubh.2015.00204

## Abbreviations

EBM, evidence-based medicine; IQ, information quality; JAMA, Journal of the American Medical Association
